# Alcohol and Tobacco Use Among Undergraduate and Postgraduate Medical Students in India: A Multicentric Cross-sectional Study

**DOI:** 10.5195/cajgh.2015.187

**Published:** 2015-02-05

**Authors:** Nidhi Goel, Vivek Khandelwal, Kapil Pandya, Atul Kotwal

**Affiliations:** Armed Forces Medical Services, India

**Keywords:** alcohol, tobacco, undergraduate medical students, postgraduate medical students, cross-sectional

## Abstract

**Background:**

Substance use among medical fraternity is a well-known phenomenon among both undergraduate (UG) and postgraduate (PG) medical students. Yet, there have been very few multi-centric studies to estimate the actual burden of this problem in this important population group in India. This study was conducted to estimate the prevalence of alcohol and tobacco use, assess the knowledge and attitudes towards this issue, and identify factors associated with substance use among UG and PG medical students in India.

**Methods:**

A pre-tested, self-report, anonymous questionnaire was administered to medical undergraduates and post graduate medical residents of eight medical colleges across India. This study used a convenience sample of medical colleges with random selection of study participants within each college for each group, UG and PG.

**Results:**

Prevalence of alcohol and tobacco use among UG students was 16.6%, 95% CI [14.5, 18.9] and 8.0%, 95% CI [6.4, 9.6], respectively, whereas prevalence was 31.5%, 95% CI [26.3, 37.0] and 14.5%, 95% CI [10.7, 18.9], respectively for PGs. For both substances, males had a higher prevalence of use compared to females in both groups (*p* < 0.001). Positive family history of substance use (*p* < 0.001 for both groups) and early age of initiation (*p* = 0.011 for tobacco; *p* > 0.05 for alcohol) were associated with a greater difficulty to quit the habit. Over 90% of study participants felt that substance use adversely affected their skills and reported not using substances prior to managing their patients.

**Conclusions:**

Since substance use is a relatively common phenomenon among UG and PG medical students in India, future prospective studies and interventions are required to better understand the pattern of substance use and reduce its prevalence.

Undergraduate (UG), as well as postgraduate (PG), medical students are exposed to daily stressors, which can lead to substance use and abuse. Substance abuse remains a covert yet well-known phenomenon among medical students and medical practitioners globally.^1^,^2^ Substances that have been documented as being used recreationally among medical students in India include alcohol, tranquillizers, and psychedelics.^3^ Recreational drug use has become more popular and may be representative behavior of the youth lifestyle; thus, increasing the need to monitor drug use trends, especially among UG and PG medical students.^1^

Substance abuse is any maladaptive pattern of substance use leading to clinically significant impairment or distress. Impairment in social and occupational functioning is often associated with substance use, which includes the inability to control use of or to discontinue use of the substance. Additionally, substance use may be associated with the development of serious withdrawal symptoms after cessation of or reduction in use for recreational or medical purposes.^4^

In the developed countries, studies have shown that alcohol impairment is one of the leading reasons for disciplinary action against physicians.^5^ However, this problem and effective interventions to reduce substance use among physicians have not been sufficiently explored in published research studies. Numerous studies conducted in the field of substance use around the world have focused mainly on the general population and adult age groups.^6^,^7^ Most of the Indian studies in this field are regional in nature, representing a large research gap which our study is aiming to address. There is a dearth of large multi-centric efforts in India to evaluate this important public health concern.^8^–^11^

Global prevalence rates of alcohol abuse disorders among adults were estimated to range from 0-16.0% with highest prevalence rates in Eastern Europe.^12^ Tobacco consumption and nicotine addiction is a major global concern, popularly known as the “brown epidemic.”^13^ The prevalence of smoking is as high as 51.0% in men (WHO Western Pacific Region) and 22.0% in women (WHO European Region) with an increasing trend in adolescent girls and boys globally.^13^

In India, alcohol use figures vary widely from 3.8% to 21.0%, with men 9.7 times more likely to regularly use alcohol as compared to women.^8^,^9^,^14^ Prevalence of smoking has been estimated as 26.0% in males and 4.0% in females. For youth, the prevalence is 19.0% and 8.3%, respectively.^15^ In previous studies, men were 25.5 times more likely than women to report regular smoking and 3.7 times more likely to regularly chew tobacco.^13^

There are few studies to date that have examined the prevalence of tobacco and alcohol use among UGs and PGs. Out of the available studies, tobacco and alcohol use prevalence was 9.0% in UGs and 7.1 in PGs.^10^,^11^,^16^ Young physicians had a prevalence of 16.7% for alcohol use.^17^

Thus, geographic variation and changing trends in substance use around the globe warrant new epidemiologic investigations that can be used to inform policy changes. Medical fraternity (UG as well as PG) is no different from the general population when it comes to substance use; however, they may be at a higher risk of substance use problems due to higher stress levels.

This study was conducted to estimate the prevalence of alcohol and tobacco use, to assess the knowledge and attitudes towards this issue, and to explore possible risk factors associated with substance use among UG and PG medical students in India. An effort was also made to gather suggestions from the fraternity for tackling this problem. Use of illegal drugs and psychoactive substances were not studied due to ethical concerns as well as the potential lack of willingness to participate in the study.

## Methods

### Participants and settings

Twelve medical colleges were recruited for participation in the study; however, four colleges did not have a sufficient sample of students for participation. The resulting 8 medical colleges participated in a multi-centric, cross-sectional survey. There were four medical colleges from Maharashtra and one each from New Delhi, West Bengal, Madhya Pradesh, and Kerala. The selection of medical colleges was convenience based, as per logistics and staff availability. For each selected college, a sample size was calculated using an estimated alcohol use prevalence of 25.0%. With alpha as 0.05 and an error margin as 7.5%, the appropriate sample size was 128. A sample of 150 final year Bachelor of Medicine, Bachelor of Surgery (MBBS) degree students and 55 PG residents was drawn from each college by simple random sampling.

Ethical clearance for the study was obtained from Institutional Ethical Committee of Armed Forces Medical College, Pune, India. Informed consent was obtained from each participant. A self-report, anonymous, pretested questionnaire covering multiple domains of substance use was used for data collection. Statistical analysis was performed using SPSS (version 14.0).

### Data Analysis

Descriptive statistics were obtained to describe basic characteristics of the study participants. Descriptive statistics were also used to estimate the prevalence of alcohol and tobacco use. Inferential statistics were used to compare male and female substance use and for differences between UG and PG for age of initiation of substance use. Age of initiation was compared amongst two groups, greater than 16 years of age and 16 years of age or younger at initiation, and then further analyzed to assess impact on difficulty in quitting the habit. Chi-Square test was used to measure the risk associated with family history of substance use and current substance use and to assess the knowledge and attitudes towards substance use. *P*-values lower than 0.05 were considered significant.

## Results

The mean age of the participating UGs was 21.5 ± 1.6 years and PGs was 29.0 ± 3.7 years. 46.2% of UG students and 72.7% of PG students were male. Table 1 shows the distribution of participant characteristics and prevalence of substance usage in each college.

Out of the 1,455 participants, males were significantly more involved in substance use as compared to females (*p* < 0.001 for alcohol as well as tobacco). Age of initiation was significantly lower in UGs as compared to PGs (*p* = 0.003) for alcohol as well as tobacco. When this age was dichotomized into 16 years or less and greater than 16 years, younger age of initiation was significantly associated with more difficulty in quitting tobacco (*p* = 0.011) but not alcohol.

Positive family history was significantly associated with increased prevalence of substance use, as shown in Table 2. Those who had a positive family history also found it more difficult to quit (*p* < 0.001). Table 3 shows the percentages of participants who attempted to quit.

‘Feeling psychologically upset’ (30.8% alcohol; 45.2% tobacco) and ‘pressure by friends’ (41.0% alcohol; 19.0% tobacco) were the most common reasons reported for resumption of the habit in those who quit but relapsed. A significant association between respondents’ “thinking substance abuse was a growing problem” and their “thinking that something should be done about it” (*p* = 0.003) was also found. Alcohol use affecting overall performance as a physician was mentioned by 65.6% of PGs.

Beverage preferences associated with alcohol and mode of tobacco consumption are depicted in Table 4. When questioned about the description of occasions when they consumed alcohol or tobacco, the highest number of participants answered ‘with close friends’ followed by ‘during parties.’ However, for the occasion ‘during exams,’ frequency of tobacco use increased more than that of alcohol. “Batch-mates” (or classmates), which refers to a cohort of students of the same entrance year in medical school, were the most common people to encourage the participants use either of the substances, followed by “seniors.”

## Discussion

Our study was conducted in 8 medical colleges across India. It revealed that prevalence of alcohol consumption for UG students and PG students was 16.6%, 95% CI [14.5, 18.9] and 31.5%, 95% CI [26.3, 37.0], respectively. Tobacco usage for UG students and PG students was found to be 8.0%, 95% CI [6.0, 9.6] and 14.5%, 95% CI [10.7, 18.9], respectively.

The former are thought to be especially vulnerable to prescription drug use due to greater access and the professional culture that favors pharmacological approaches to the management of occupational stress.^3^,^9^ Studies have shown an alarming increase in drug, alcohol, and tobacco consumption among doctors in the latter half of 20^th^ century globally.^11^ Personal use of addictive substances by doctors has the potential to jeopardize their professional performance, as well as care of patients with addictions,^18^ thus, it is important to investigate this area of concern.

82.5% medical students in previously published Irish study were current alcohol consumers,^18^ whereas the prevalence was only 16.6% in our study. For resident doctors, the prevalence of 31.5% in our sample was higher than 16.7% as brought out in previous studies on young Indian physicians.^17^ The prevalence of alcohol use among our study participants is higher when compared with prevalence among adult Indian general population, which ranges between 3.8% to 4.5%.^8^,^12^

For medical students, the prevalence of current tobacco usage has been previously reported to be 10% in the Indian population^17^ and 15.3% in Western populations.^18^ In our study, the prevalence of current tobacco usage is 8% and 14.5% among medical students and doctors, respectively, which is almost equal to the national medical fraternity figures. The prevalence of tobacco use in any form is much lower in medical students (17.5%) as compared to students in general (21.6%).^19^ Tobacco was consumed in smoking form by the majority of its users, which is consistent with national as well as global trends.

Our study highlights the finding that for medical fraternity, the overall prevalence of tobacco usage is lower than the national figures. This is true in some other countries, including China.^20^ In countries like the United States of America, Greece, Japan, and Australia, the trends of substance use in medical fraternity follows the national prevalence rates.^20^ The overall low prevalence of tobacco usage may be attributed to bans on smoking in campuses in the government medical colleges.

For both substances, males had a higher prevalence of consumption compared to females. This was true for medical students, as well as resident doctors (*p* = 0.001). This trend is similar to other published studies conducted in India and across the world.^1^,^20^ Though alcohol use rates have been increasing in females according to some studies,^18^ no such trends were found in this study due to its cross sectional design. Future prospective studies may be able to more thoroughly examine temporal trends in substance use.

One of the interesting findings of this study is that medical students and doctors who had a positive family history of substance use had higher prevalence of substance use. Though this factor is not commonly analyzed in previously published investigations, this finding is very important for India, where family values greatly influence the behaviors of younger people as reported in previous Indian study.^3^ Thus, families can potentially play a role in reducing substance consumption in the medical community.

Another factor which is scarcely reported in the literature on substance use among medical professionals is the age of initiation of substance usage. In our study, we found that current UG students are beginning the usage of alcohol or tobacco much earlier than current PG students (*p* = 0.01). Also, for tobacco, earlier age of initiation (less than 16 years) was associated with difficulty in quitting. This means that the efforts to educate and counsel the medical students on problems associated with substance use must start much earlier than previously thought, preferably at the very beginning of their medical curriculum.

Among the reasons for initiation, “curiosity” was the most common reason reported by the respondents in our study. This response is consistent with most studies on this subject.^8^–^11^ The implication is that if the concepts of neuro-physiological mechanisms involved in substance use and addiction are explained to the students at the time of entry into the medical colleges, the curiosity factor and the experimentation tendency might be reduced. Other reasons for initiation of alcohol use were ‘pleasure’ and ‘stress/anxiety relief.’^1^

The individuals most commonly encouraging or influencing initiation of substance use were ‘batch-mates.’ Also, for most of those who tried to quit, it was their friends that pressured them to relapse. Thus, if we are able to reduce substance use rate in a small group of students, they potentially would be able to influence others to change their behaviors and reduce substance use. However, a large number of students reported that no one encouraged them, indicating that a significant group of students were self-motivated to initiate the habit. Thus, this group can particularly benefit from Behavior Change Communication (BCC) activities.

An interesting finding is that among doctors using any substance, 92.0% – 95.0% had never consumed the respective substance prior to managing their patients or conducting a medical/ surgical procedure, suggests that respondents understand that substance use may influence their professional performance.

## Strengths and limitations

The main limitation of this study was the convenience sampling method used to select the medical colleges. The other limitation was a low response rate in a few of the medical colleges or lack of willingness to answer questions related to alcohol consumption. Additionally, cross-sectional nature of this study did not allow us to make conclusions about substance use trends over time, limitation that will be addressed by future research studies.

The key strength of this study was its multi-centric approach, since medical colleges from different parts of the country were involved. Also, this study examines various risk factors and correlates for substance use in UGs as well as PGs, which can potentially guide counseling efforts or other interventions. This study provides a foundation for future prospective studies can be conducted regarding the role of BCC approach targeted at specific points in a medical student’s career.

## Figures and Tables

**Table 1:** Distribution of alcohol and tobacco use among participants stratified by college and student group

**Medical** **College**	**UG Students**			**PG Residents**		

	N	**Prevalence (95% CI)**		**Prevalence (95% CI)**		N
	
		Alcohol	Tobacco	Alcohol	Tobacco	
**1**	100*	16(9.4,24.6)	11(5.6,18.8)	41*	29.3(16.1,45.5)	17.1(7.1,32)
**2**	151	38.4(30.6,46.6)	21.9(15.5,29.2)	35*	40(23.8,57.8)	22.9(10.4,40.1)
**3**	151	17.2(11.5,24.2)	7.9(4.1,13.4)	35*	40(23.8,57.8)	22.9(10.4,40.1)
**4**	148	4.1(1.5,8.6)	0	50	22(11.5,35.9)	0
**5**	150	7.3(3.7,12.7)	2.7(0.7,6.6)	50	30(17.8,44.6)	8(2.2,19.2)
**6**	151	17.2(11.5,24.2)	7.9(4.1,13.4)	0*	0	0
**7**	148	4.1(1.5,8.6)	0	50	22(11.5,35.9)	0
**8**	145	28.3(21.1,36.3)	13.1(8.0,19.7)	50	42(28.1,56.8)	36(23,50.8)
**TOTAL**	1,144	16.6(14.5,18.9)	8(6.,9.6)	311	31.5(26.3,37.0)	14.5(10.7,18.9)

**Grand Total**	N=1,455 Total prevalence among med fraternity: Alcohol – 19.8(17.8, 21.9); Tobacco – 9.4(8.0, 11.0)					

*Note*.

* indicates that the number of participants enrolled were less than the required sample size

**Table 2:** Association of a positive family history with consumption of substances among UGs and PGs

**Family History**		**Self-consumption**		*p*-value*

		**Yes**	**No**	
**Alcohol**	+	156	245	< 0.001

	−	132	922	

**Tobacco**	+	52	171	< 0.001

	−	84	1,148	

*Note*.

* Based off Chi-square analyses.

**Table 3:** Proportion of participants who tried to quit the habit

	**Alcohol**	**Tobacco**
No answer	11 (3.8%)	12 (8.8%)
No	237 (82.3%)	82 (60.3%)
Yes	40 (13.8%)	42 (30.9%)

**Table 4:** Alcohol types and tobacco modes used for consumption

**Type of Substance**	**N**
Alcohol	
Beer	226
Vodka	175
Whisky	162
Rum	100
Other	146
Total	288 (19.8%)
Tobacco	
Smoking	120
Chewing	3
Sniffing	1
Total	136 (9.3%)

*Note.* Discrepancy in totals is due to multiple responses for some options and no response by some users.
